# Factors Influencing Patients’ Choice of a Joint Replacement Surgeon

**DOI:** 10.7759/cureus.99465

**Published:** 2025-12-17

**Authors:** Whisper Grayson, Garrett Basich, Daniel Schmitt, Nicholas Brown

**Affiliations:** 1 Orthopaedic Surgery and Rehabilitation, Loyola University Medical Center, Maywood, USA; 2 Orthopaedic Surgery, Idaho College of Osteopathic Medicine, Meridian, USA

**Keywords:** elective orthopedic surgery, orthopedic surgery, patient selection, questionnaire survey, total joint arthroplasty

## Abstract

Introduction: Technology has undergone rapid growth over the past several decades, with the internet and social media becoming more available to individuals. Thus, the medical field has entered a new era where patients have increased access to information about their physicians. This has led to an evolving area of research seeking to determine the impact certain factors may have when patients choose where to receive their medical care. The primary aim of this study was to evaluate the importance of certain factors to new patients presenting to an orthopedic adult reconstruction clinic.

Methods: New patients presenting to an orthopedic adult reconstruction clinic at an academic medical center in a large metropolitan area from August 2024 to February 2025 were invited to complete an anonymous survey, with a total of 50 participants ultimately included. The survey consisted of demographic questions and Likert-type questions assessing selection factor importance. The importance of each factor was scaled according to five categories: (1) least important, (2) somewhat important, (3) moderately important, (4) very important, and (5) most important.

Results: Factors considered most important to the study participants included the reputation of the institution (mean: 4.1, standard deviation (SD): 0.9), if the surgeon had been in practice for more years (mean: 3.8, SD: 1.0), and if the provider/hospital was in the patient’s insurance network (mean: 3.7, SD: 1.5). There were no significant differences between responses when comparing the patients ≥65 years of age and those <65 years.

Conclusions: Institutional reputation, the number of years the surgeon has been in practice, and insurance coverage were important factors to patients when selecting an adult reconstruction surgeon. While not significant, younger patients found online reviews and social media presence of their surgeon to be more important compared to older patients.

## Introduction

With the world becoming more connected than ever through various media platforms, the medical field has entered a new era in which patients have unprecedented access to information about their physicians [1,2]. Prior studies assessing the impact of internet use in the United States have found that approximately 80% of people seeking medical care refer to the internet for information [3]. Access to online reviews, physician backgrounds, and social media platforms allows patients to take a more active role in where they choose to go for their medical care [4-6]. Consequently, a growing body of research is exploring how patients make choices when selecting healthcare providers.

There has been a recent research emphasis on evaluating what factors are important to patients when choosing where to receive their medical care. Prior studies have found that institutional reputation, the number of years the surgeon has been in practice, and whether the practice is in-network for insurance coverage are important factors to patients [7,8]. Soft skills, including empathy, compassion, and active listening, also proved to be significant factors in a patient’s decision when selecting a surgeon [9]. With the rapidly evolving world of social media and the internet, studies have sought to evaluate the significance these platforms play in physician selection. While some studies have found that online advertisement and social media presence did not hold consequential influence over a patient’s decision, other studies have demonstrated an increasing reliance on these modes among younger patients [8,10,11].

Recent studies within orthopedic subspecialities have contributed to this current literature, highlighting the selection factors patients find important, including physician credentials and reputation [8,10]. Despite these prior studies, there remains room to further investigate this topic within the setting of an orthopedic adult reconstruction clinic. The primary aim of this study was to invite new patients presenting to an adult reconstruction clinic for elective total joint arthroplasty to complete a voluntary and anonymous survey assessing patient demographics, social media/internet usage, and the importance of various selection factors when deciding upon their surgeon. We primarily aimed to determine which surgeon- and institution-related factors are most influential in patient decision-making. A secondary aim was to compare the relative importance of these factors between patients younger than 65 and those 65 or older.

## Materials and methods

Study design

Following institutional review board (IRB) approval, a prospective survey study was conducted at a tertiary academic medical center in a large metropolitan area from August 2024 to February 2025. All new patients presenting to an adult reconstruction clinic were invited to complete an anonymous survey. Inclusion criteria included new patients who presented to one of two fellowship-trained arthroplasty surgeons within the aforementioned time frame. Exclusion criteria included any previously established patients. Participants were given a cover letter explaining the premise of the study prior to the completion of the survey.

The survey consisted of three parts and was adapted from a validated instrument. The first included demographic questions, assessing age, gender, health insurance source, highest level of education, and whether they received a referral. The second category assessed their frequency of internet (WebMD.com and HealthGrades.com) and social media (Facebook, Instagram, Twitter, and/or TikTok) utilization. The third category evaluated the importance of various factors patients may have considered when choosing an adult reconstruction surgeon. The importance of each factor was scaled according to five categories: (1) least important, (2) somewhat important, (3) moderately important, (4) very important, and (5) most important. Lastly, the final category assessed post-selection satisfaction, rating each question according to the following categories: strongly disagree, disagree, neutral, agree, and strongly agree.

Data analyses

Means and standard deviations (SDs) are provided for continuous variables, while percentages and sample size are provided for categorical variables. Each Likert-type response in the third category was converted to a numerical variable and reported as a mean with standard deviation. These means were compared between patients ≥65 years of age and those <65 years using a two-sample t-test, with an alpha level of 0.05 used for statistical significance.

Patient demographics

A total of 50 new patients during the enrollment period opted to be included in the study. Twelve new patients opted not to be included in the study, giving an overall enrollment rate of 80.6%. A majority (58%) of the patients were female, and the average age was 63.4 years (range: 42-83). A majority (44%) of the patients had employee insurance, followed by government (30%) and private (16%). A majority (90%) of the patients had received a referral (Table 1).

**Table 1:** Baseline demographics of the study population (N=50) Data are represented as means ± standard deviations for continuous variables and percentages (%) and sample size (N) for categorical variables. GED: General Educational Development

Characteristics	Study cohort (N=50)
Average age (years)	63.4 (10.1)
Gender	
Female	58% (29)
Male	42% (21)
Health insurance	
Private	16% (8)
Employee	44% (22)
Government	30% (15)
Uninsured	0% (0)
Other	10% (5)
Highest level of education	
Graduate or professional degree	14% (7)
College degree	28% (14)
Some college, no degree	22% (11)
High school or GED	24% (12)
No degree	12% (6)
Referral	
Yes	90% (45)
No	10% (5)

## Results

Internet and social media usage

Most patients infrequently used the internet and social media. The most commonly used source was Facebook, with 20% of patients reporting daily use. Medical websites were infrequently used, with a majority of patients (64% and 84%, respectively) denied having ever used WebMD.com or HealthGrades.com (Table 2).

**Table 2:** Frequency of social media and internet utilization among patients (N=50) Data are represented as percentages (%) and sample size (N).

Platform/website	Frequency of use			
	Never	Occasionally	Frequently	Daily
Facebook	40% (20)	20% (10)	20% (10)	20% (10)
Twitter	82% (41)	6% (3)	10% (5)	2% (1)
Instagram	70% (35)	22% (11)	4% (2)	4% (2)
TikTok	90% (45)	8% (4)	0% (0)	2% (1)
WebMD.com	64% (32)	34% (17)	2% (1)	0% (0)
HealthGrades.com	84% (42)	16% (8)	0% (0)	0% (0)

Selection factors

**Figure 1:**
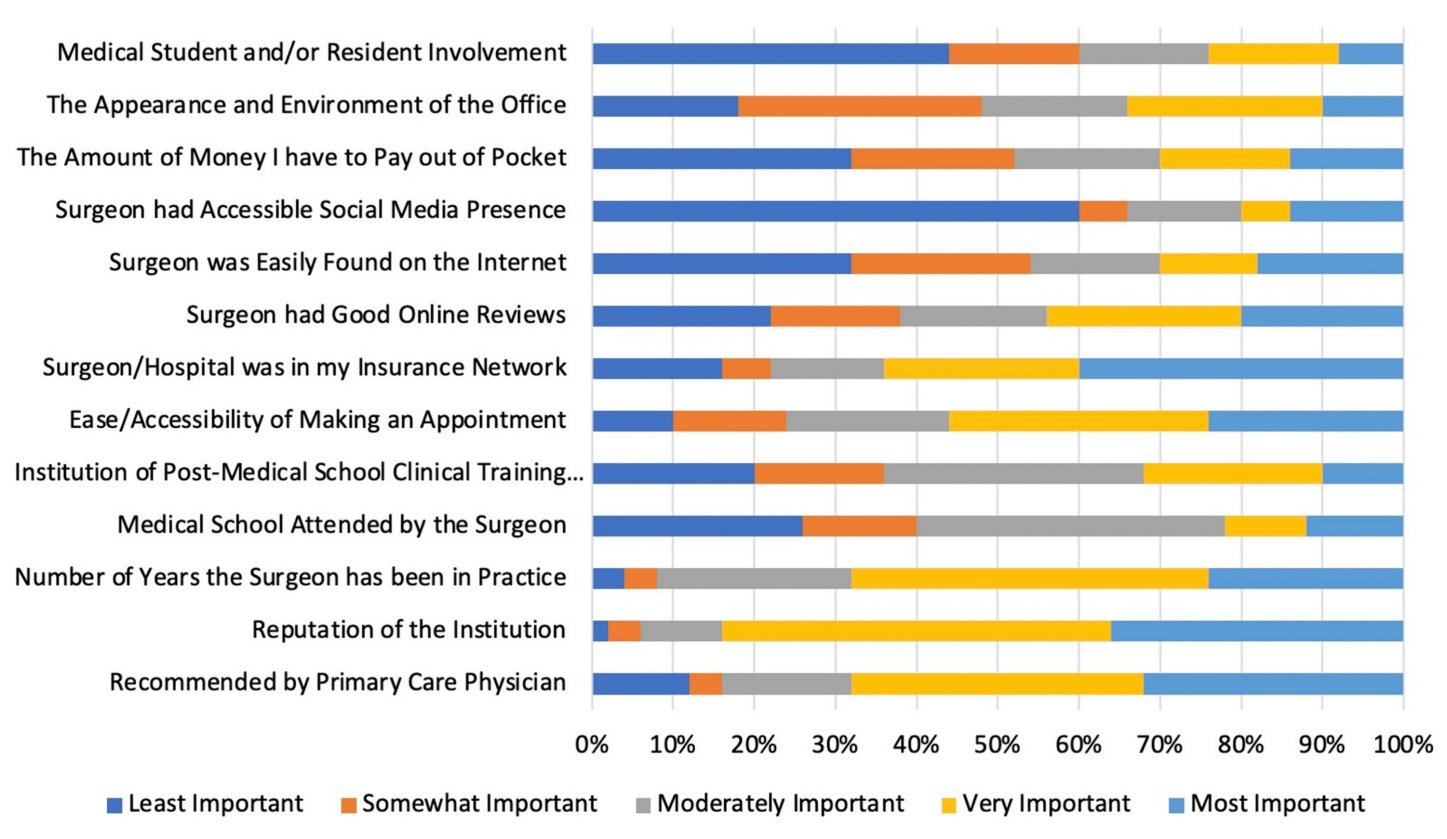
Importance of selection factors among new patients

**Figure 2:**
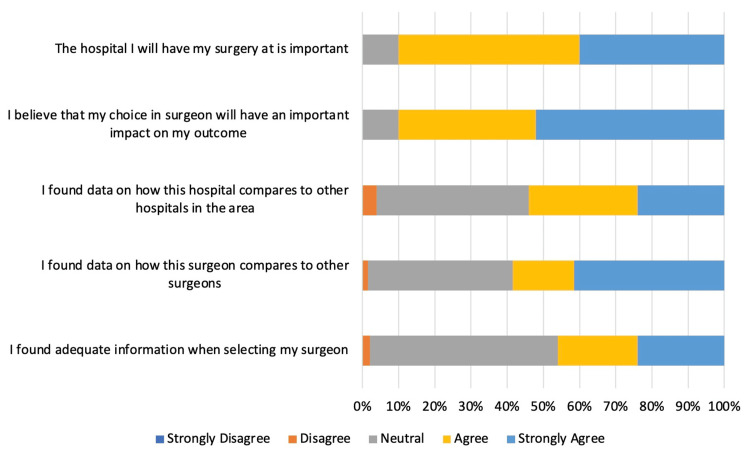
Post-selection statement agreement among patients

Factors considered most important to the study participants included the reputation of the institution (mean: 4.1, SD: 0.9), the number of years the surgeon has been in practice (mean: 3.8, SD: 1.0), and if the provider/hospital was in the patient’s insurance network (mean: 3.7, SD: 1.5). Less important considerations included whether the surgeon had a social media presence (mean: 2.1, SD: 1.5) and whether there was resident or medical student involvement in the clinic (mean: 2.3, SD: 1.4) (Figure 1, Table 3). Post-selection, patients felt they were able to adequately find information online to make an informed decision when choosing a surgeon and hospital (Figure 2).

**Table 3:** Importance of selection factors among patients (N=50) Data are represented as means ± SD. SD: standard deviation

Selection factor	Mean (SD)
Recommended by primary care physician	3.7 (1.3)
Reputation of the institution	4.1 (0.9)
Number of years the surgeon has been in practice	3.8 (1.0)
Medical school attended by the surgeon	2.7 (1.3)
Institution of post-medical school clinical training (residency and fellowship) completed by the surgeon	2.9 (1.3)
Easy/accessibility of making an appointment	3.5 (1.3)
Surgeon/hospital was in my insurance network	3.7 (1.5)
Surgeon had good online reviews	3.0 (1.5)
Surgeon was easily found on the internet	2.6 (1.5)
Surgeon had accessible social media presence	2.1 (1.5)
The amount of money I have to pay out of pocket	2.6 (1.4)
The appearance and environment of the office	2.8 (1.3)
Medical student and/or resident involvement	2.3 (1.4)

Factors by age group

When comparing the selection factor responses between patients ≥65 years of age and those <65 years, there were no significant differences between the responses. There was, however, a non-statistically significant trend toward patients under 65 years putting more weight on the number of years the surgeon had been in practice (4.0±0.8 versus 3.6±1.1, p=0.098), online reviews (3.4±1.4 versus 2.7±1.4, p=0.079), and whether they had a social media presence (2.5±1.7 versus 1.7±1.2, p=0.058) (Table 4).

**Table 4:** Comparison of selection factors between patients ≥65 years of age (n=26) and those <65 years (n=24) Data are represented as means ± standard deviations. Two-sample t-tests were used to evaluate the data, with a p-value of 0.05 used to determine significance.

Selection factor	≥65 years	<65 years	T-value	P-value
Recommended by primary care physician	3.5 (1.4)	3.9 (1.1)	-1.033	0.307
Reputation of the institution	4.0 (1.0)	4.3 (0.8)	-0.986	0.329
Number of years the surgeon has been in practice	3.6 (1.1)	4.0 (0.8)	-1.690	0.098
Medical school attended by the surgeon	2.4 (1.3)	3.0 (1.3)	1.471	0.148
Institution of post-medical school clinical training (residency and fellowship) completed by the surgeon	2.6 (1.2)	3.1 (1.3)	-1.442	0.156
Easy/accessibility of making an appointment	3.2 (1.4)	3.7 (1.1)	-1.327	0.191
Surgeon/hospital was in my insurance network	3.7 (1.6)	3.6 (1.4)	0.161	0.873
Surgeon had good online reviews	2.7 (1.4)	3.4 (1.4)	-1.797	0.079
Surgeon was easily found on the internet	2.4 (1.4)	2.8 (1.6)	-0.968	0.338
Surgeon had accessible social media presence	1.7 (1.2)	2.5 (1.7)	-1.943	0.058
The amount of money I have to pay out of pocket	2.9 (1.5)	2.3 (1.4)	1.467	0.148
The appearance and environment of the office	2.6 (1.2)	3.0 (1.4)	-0.944	0.350
Medical student and/or resident involvement	2.1 (1.3)	2.5 (1.5)	-0.872	0.387

## Discussion

In this study, we sought to identify factors that were important to patients when selecting an orthopedic adult reconstruction surgeon. Our results demonstrated an increased preference for referral, institutional reputation, and insurance coverage by patients when selecting their medical provider. Factors such as the social media presence of their surgeon were less important. Patients also felt that they were able to find adequate information online about their surgeon and the hospital prior to deciding where they would seek their care.

Our study aligns with current literature by seeking to identify what factors are important to patients when choosing where to receive their medical care, especially in an era where there is easy access to information on the internet. A similar survey study by Gusho et al. evaluated important factors for new patients presenting to an orthopedic oncology clinic [10]. Their study found that patients placed emphasis on the reputation of the hospital, as well as the number of years the surgeon has been in practice [10]. Furthermore, their study divided patients into two groups by age: those under 40 and those over 40 years old [10]. The younger age group placed a statistically significantly higher importance on social media and internet presence of their surgeon when compared to the older age group [10]. When compared to our study, we similarly found institutional reputation and the number of years the surgeon had been in practice to be important factors. Contrastingly, we did not find significantly higher preference with regard to internet or social media preference when comparing age groups. We did, however, have a higher cutoff point for age due to our practice seeing a higher-aged population. This likely impacted the significance placed on internet or social media influence.

Our study population overall reported a very low frequency of social media and internet usage. This aligns, however, with current statistics evaluating the usage of technology across various age groups. While trends have demonstrated an increasing reliance upon technology over the past couple of decades, older age groups still tend to have reduced usage when compared to their younger counterparts [12]. A recent survey by Pew Research Center found that only 75% of adults over 65 years use the internet, compared to 99% between 18 and 29 years, 98% between 30 and 49 years, and 96% between 50 and 64 years [12]. Furthermore, within the 65 and older group, only 45% report using social media and 61% report having a smartphone [12]. Thus, our patient population reporting an overall low usage of the internet and social media is not unexpected. If these trends continue to increase, however, we can expect increasing usage within this age group in the future.

Another prospective questionnaire study performed by Manning et al. included 231 patients who presented to a single spine surgeon [7]. Their results demonstrated board certification, in-network provider status, and bedside manner to be important factors in their provider decision [7]. Additional studies have supported these findings, where surgeon reputation and experience stand out as important selection factors, as well as the location and reputation of the hospital [9,13]. One study by Marshall et al. found that these factors were so important that patients were willing to wait 10 months for a total knee arthroplasty to have their surgeon of choice [14].

In the growing age of technology, patients have an expanding database of information on physicians and medical centers [1-3]. This allows them to have increased autonomy over their decision of where to seek care. As physicians and hospitals seek to understand what patients are looking for in their physicians, it is important to consider not only the in-office experience but also what information is available online. In the clinical setting, friendliness, appropriate bedside manner, and limited wait times are important to patients [9]. The study by Manning et al. found that 92% of patients felt that 30 minutes or less should pass between check-in and seeing their surgeon while in the clinic [7]. While this is a difficult factor to control, given the variability of clinics, seeking to minimize wait times will improve patient satisfaction [7]. Furthermore, while most patients seem to put less weight on internet or social media presence, they do value the education/training of the surgeon and the hospital reputation [10,14]. Thus, having this information readily available online can help inform a patient while they are making their decision. Lastly, hospital proximity and insurance coverage are non-modifiable factors but are still important to keep in mind when considering the location of a surgical practice, the proximity of other available practices, and the area’s patient population.

The results of this study must be considered within its limitations, including the inherent risk of recall bias and response set bias associated with a survey study. Further limitations associated with a survey study include the data being self-reported by patients, as well as the potential for respondent fatigue leading to rushed answers. The survey was designed to be brief; however, this limited the potential for inaccuracies or repetitive answers by the participants. It is also possible that the people who choose to answer a survey may be systematically different from those who decline to fill out the survey, thereby skewing the data. It is important to keep in mind that these findings reflect patterns observed within this patient population and may not generalize to other settings or age groups. Furthermore, the low patient population limits the generalizability of this study. Finally, we did not control for the impact of demographic variables and comorbidities. Nonetheless, this study contributes to the current literature on what patients consider when choosing a joint replacement surgeon.

## Conclusions

In this study, we found that a referral, the institution’s reputation, the number of years the surgeon has been in practice, and insurance coverage were important factors to patients when selecting an adult reconstruction surgeon. While not significant, younger patients found online reviews and social media presence of their surgeon to be more important compared to their elders.
